# Wnt5a through Noncanonical Wnt/JNK or Wnt/PKC Signaling Contributes to the Differentiation of Mesenchymal Stem Cells into Type II Alveolar Epithelial Cells *In Vitro*

**DOI:** 10.1371/journal.pone.0090229

**Published:** 2014-03-21

**Authors:** Airan Liu, Song Chen, Shixia Cai, Liang Dong, Le Liu, Yi Yang, Fengmei Guo, Xiaomin Lu, Hongli He, Qihong Chen, Shuling Hu, Haibo Qiu

**Affiliations:** 1 Department of Critical Care Medicine, Zhongda Hospital, School of Medicine, Southeast University, Nanjing, P. R. China; 2 State Key Laboratory of Natural Medicines, School of Life Science and Technology, China Pharmaceutical University, Nanjing, P. R. China; Rutgers - New Jersey Medical School, United States of America

## Abstract

The differentiation of mesenchymal stem cells (MSCs) into type II alveolar epithelial (AT II) cells is critical for reepithelization and recovery in acute respiratory distress syndrome (ARDS), and Wnt signaling was considered to be the underlying mechanisms. In our previous study, we found that canonical Wnt pathway promoted the differentiation of MSCs into AT II cells, however the role of the noncanonical Wnt pathway in this process is unclear. It was disclosed in this study that noncanonical Wnt signaling in mouse bone marrow–derived MSCs (mMSCs) was activated during the differentiation of mMSCs into AT II cells in a modified co-culture system with murine lung epithelial-12 cells and small airway growth media. The levels of surfactant protein (SP) C, SPB and SPD, the specific markers of AT II cells, increased in mMSCs when Wnt5a was added to activate noncanonical Wnt signaling, while pretreatment with JNK or PKC inhibitors reversed the promotion of Wnt5a. The differentiation rate of mMSCs also depends on their abilities to accumulate and survive in inflammatory tissue. We found that the Wnt5a supplement promoted the vertical and horizontal migration of mMSCs, ameliorated the cell death and the reduction of Bcl-2/Bax induced by H_2_O_2_. The effect of Wnt5a on the migration of mMSCs and their survival after H_2_O_2_ exposure were partially inhibited with PKC or JNK blockers. In conclusion, Wnt5a through Wnt/JNK signaling alone or both Wnt/JNK and Wnt/PKC signaling promoted the differentiation of mMSCs into AT II cells and the migration of mMSCs; through Wnt/PKC signaling, Wnt5a increased the survival of mMSCs after H_2_O_2_ exposure *in vitro*.

## Introduction

Despite extensive studies on the pathophysiology and treatment of acute respiratory distress syndrome (ARDS), the mortality remains at 34–44 percent [1]. The damage of the alveolar epithelial barrier, which mainly consists of type I and type II alveolar epithelial cells, is the main pathological characteristic and the therapeutic target of ARDS. AT II cells play an important role in pulmonary physiology and pathology because they are capable of synthesizing and secreting alveolar surfactant to reduce surface tension and prevent the collapse of the alveoli and because they may differentiate into the type I alveolar epithelial cells (AT I cells) to serve as progenitor cells for the reepithelization of impaired alveoli [2]. Thus, promoting the regeneration and repair of injured AT II cells is critical for patient recovery from ARDS.

Recently, the potential of multipotent mesenchymal stem cells (MSCs) in the treatment of ARDS has been revealed in several investigations [3]–[6]. It was found that MSCs, which can differentiate into AT II cells *in vitro* and *in vivo*, were involved in the repair of the alveolar epithelium in ARDS [7]–[10]. However, their engraftment and differentiation rates in injured lungs were limited [5], and further exploration is needed to improve these rates. Thus, it is necessary to explore the mechanisms underlying the differentiation of MSCs into AT II cells and their migration to injured lung tissue.

Wnt pathway is considered to be important in regulating mechanisms for the proliferation, development, differentiation of cells and organisms and can be divided into canonical and noncanonical Wnt pathway. In our previous study, we found that the activation of the canonical Wnt pathway promoted the differentiation of mice bone marrow-derived MSCs (mMSCs) into AT II cells based on a model for the differentiation of mice bone marrow derived MSCs (mMSCs) into AT II cells with indirectly co-cultured with murine lung epithelial (MLE)-12 cells plus small airway growth media (SAGM) [10]. However, the role of noncanonical Wnt pathway which functions independent of the accumulation of β-catenin and is as critical as canonical wnt signaling in modulating differentiation, proliferation, migration of cells, in the differentiation of MSC into AT II cells have not been well explored. In the noncanonical Wnt pathway, Wnt ligand such as Wnt5a induces calcium influx through Dvl, followed by the phosphorylation of the downstream Calmodulin-dependent protein kinase (CaMK) or Protein kinase C (PKC), which regulates target gene expression through the activation of the nuclear factor of activated T cells (NFAT). JNK is another downstream effector of Wnt5a that activates activator protein 1 (AP-1), thereby regulating PCP (planar cell polarity) signaling [11], [12].

Several recent studies have shown that noncanonical Wnt signaling has critical effects on the differentiation of MSCs, which express a number of ligands, receptors and pathway inhibitors [13]. Boland, et al. [14] found that Wnt5a could promote MSC differentiation into osteoblasts. Topol, et al. [15] also found that Wnt5a hinders the chondrogenic lineage commitment of MSCs, promotes chondrocyte differentiation, and delays chondrocyte maturation into hypertrophic stages. Additionally, the overexpression or shortage of noncanonical Wnt signaling could induce dysplasia of the alveolar epithelium [16], therefore we believe that noncanonical Wnt signaling may have a critical effect on the differentiation of MSCs into AT II cells, although this has not yet been investigated.

The aims of our study were to explore the role and underlying mechanisms of noncanonical Wnt signaling in the differentiation of mice bone marrow derived (mMSCs) into AT II cells according to the detection of the some AT II cells related markers as well as the ability of mMSCs to survive under oxidative stress conditions and migrate to injured lung tissue *in vitro*. The latter two properties can promote the localization and survival of MSCs in impaired lung tissue, thus indirectly influencing the differentiation of MSCs into AT II cells.

## Materials and Methods

### Ethics statement

All experimental procedures with animals used in the present study were complied with the National Research Council's guidelines and had been given prior approval by Care of Experimental Animals Committee of the Southeast University (approval ID: 2011-0122).

### Cell culture

mMSCs and MLE-12 cells were used in the present study. mMSCs, obtained from Cyagen Biosciences Inc. (Guangzhou, China), were isolated from the bone marrow of C57BL/6 mice. The cells were verified as mesenchymal stem cells according to the identification of cell surface phenotypes (CD34+, CD44+, CD29+, SCA-1+ and CD117−) and the multipotent differentiation potential along the adipogenic, osteogenic, and chondrogenic lineages offered by the supplier [10]. MLE-12 cells, purchased from American Type Culture Collection (ATCC, Manassas, VA, USA), and mMSCs were cultured in Dulbecco's modified Eagle media/nutrient F-12 (DMEM/F12) 1∶1 mixture (Thermo Scientific Hyclone, Beijing, China) supplemented with 2% (for MLE-12 cells) or 10% (for mMSCs) fetal bovine serum (FBS; Wisent Inc., St-Bruno, Quebec, Canada), 100 U/ml penicillin and 100 µg/ml streptomycin (Thermo Scientific Hyclone) in a humidified 5% CO_2_ incubator at 37°C. The culture media was changed every 3 days, and the cells were passaged when they reached 90% confluency.

### The differentiation of mMSCs into AT II cells

According to our previous study [10], a co-culture system with MLE-12 cells and SAGM (Lonza Group Ltd., Basel, Swizerland) was adopted for driving the differentiation of mMSCs into AT II cells. Briefly, 1×10^4^ mMSCs and MLE-12 cells in 1.5 ml or 1 ml DMEM/F12 media supplemented with 10% FBS were, respectively, seeded in the lower or upper chambers of Transwell inserts (0.4-µm pore size, 4.5 cm^2^, Corning, Inc., Corning, NY, USA) to establish the co-culture system. After mMSCs reached 80% confluency three days later, the culture media was replaced with SAGM, which consisted of small airway epithelial basal media and supplements, including 0.5 mg/ml bovine serum albumin, 30 µg/ml bovine pituitary extract, 0.5 µg/ml hydrocortisone, 0.5 ng/ml epithelial growth factor, 0.5 µg/ml epinephrine, 5 µg/ml insulin, 6.5 ng/ml triiodothyronine, 10 µg/ml transferring and 0.1 ng/ml retinoic acid, for another 7 days. To investigate the role of noncanonical Wnt signaling in mMSC differentiation, 500 ng/ml Wnt5a (R&D Systems, Minneapolis, MN, USA), in the presence or absence of 5 µmol/L SP600125 (Merck Biosciences, Darmstadt, Germany), a blocker of JNK, or 2.5 µmol/L GF109203X (Enzo Life Science, Farmingdale, NY, USA), a blocker of PKC were added to the co-cultured conditions. After differentiation, the inserts were removed, and mMSCs were harvested for western blotting and quantitative real-time PCR (qRT-PCR) analysis.

### Cell proliferation and viability assay

To evaluate the effect of the noncanonical Wnt pathway on the proliferation and tolerance to H_2_O_2_-induced oxidative stress in mMSCs, 1×10^3^ cells in 100 µl DMEM/F12 supplemented with 2% FBS were seeded into flat-bottomed 96-well culture plates. When the cell confluency reached 30–40% (for the proliferation test) or 75–85% (for the tolerance to H_2_O_2_ test), the growth media were supplemented with certain concentrations of Wnt5a, SP600125 or GF109203X for the indicated time in the absence or presence of H_2_O_2_. Then, the cell number was evaluated using a modified 3-(4, 5-dimethylthiazol-2-yl)-2, 5-diphenyltetrazolium (MTT) assay (Sigma, St. Louis, MO, USA), and the absorbance of resulting formazan was measured at 570 nm (630 nm as a reference).

### Cell migration assay

The vertical and horizontal migrations of mMSCs were determined through a Transwell migration assay or wound healing assay, respectively. In the vertical migration test, 1×10^4^ mMSCs in 200 µl serum-free DMEM/F12 containing 500 ng/ml Wnt5a or 500 ng/ml Wnt5a plus 5 µmol/L SP600125 or 2.5 µmol/L GF109203X were loaded into the Transwell inserts (6.5 mm diameter and 8 µm pore size, Corning, Inc.). Then, either 600 µl DMEM/F12 supplemented with 10% FBS or conditioned media of lung tissue obtained from normal or ARDS mice was added in the 96-well culture plates below the inserts. The conditioned media of mice lung tissue were obtained in accordance with our previous study [10]. Briefly, male C57BL/6 mice between 8 to 10 weeks of age were randomly intratracheally administrated with lipopolysaccharide (LPS, Escherichia coli strain 0111:B4, Sigma-Aldrich) dissolved in sterile phosphate-buffered saline (PBS) (2 mg/kg) or the same amount of PBS after anaesthesia with butaylone (Sigma-Aldrich) intraperitoneal administration. After being sacrificed 24 hours later, their right lung lobes were harvested then cut into small pieces, which were incubated with 2% FBS-DMEM/F12 media for another 6 hours to acquire the conditioned media, and the left lung lobes were used to detect the expression of Wnt5a using western blotting. After 10 hours of migration in a humidified CO_2_ incubator at 37°C, the average number of migratory cells were detected by counting the cells stained with crystal violet (Beyotime Institute of Biotechnology, Haimen, China) in five fields under a microscope (×200).

In the wound healing assay, after the mMSCs reached 90% confluency in 96-well culture plates, the cell monolayer was scraped in a straight line to create a “scratch” with a 10 µl pipet tip. The original culture media and the debris were replaced with 2% FBS-DMEM/F12 media supplemented with 500 ng/ml Wnt5a or 500 ng/ml Wnt5a plus 5 µmol/L SP600125 or 2.5 µmol/L GF109203X. The cells were incubated in a humidified 5% CO_2_ incubator at 37°C for 12 hours, and images under a phase-contrast microscope were acquired for further measurement of the intervals.

### Western blotting analysis

Total protein and nucleoprotein from cells and lung tissue were extracted using RIPA lysis buffer (Beyotime Institute of Biotechnology) or a nuclear protein extraction kit (Beyotime Institute of Biotechnology) supplemented with 1 mmol/L PMSF, 1 mmol/L NaF and 1 mmol/L Na_3_VO_4_, respectively, according to the manufacturer's instructions. Following separation by 10 or 12% sodium dodecyl sulfate-polyacrylamide gel electrophoresis (SDS-PAGE), the proteins were electro-transferred to PVDF membranes (Millipore, Bedford, MA, USA), which were then blocked for 1 hour at room temperature and incubated at 4°C overnight with primary antibodies against β-catenin, p-CamK II, p-SAPK/JNK (Thr183/Tyr185), SAPK/JNK, p-PKCα/β II (Thr638/641), p-PKC (pan) (β II Ser660), Wnt5a/b (Cell Signaling Technology, Beverly, MA, USA), CamK II β/γ/δ, PKC pan (Bioworld Technology inc., MN, USA), pro-surfactant protein C (pro-SPC) (Millipore), Bax, Bcl-2 and β-actin (Santa Cruz Biotechnology, Inc., Santa Cruz, CA, USA). On the following day, the immunoreactive bands were detected with a chemiluminescence imaging system (ChemiQ 4800mini, Ouxiang, Shanghai, China) after incubation with a horseradish peroxidase-conjugated secondary antibody (Zhongshan Golden Bridge Biotechnology Co., Ltd., Beijing, China) for 1 hour at room temperature.

### Quantitative real-time polymerase chain reaction

The levels of surfactant protein (SP) B, SPC, SPD and aquaporin (AQP) 5 mRNA in mMSCs after differentiation into AT II cells were analyzed via qRT-PCR. Total RNA was extracted using TRIzol reagent (Takara Bio, Inc., Kyoto, Japan), according to the manufacturer's instructions. Then, an equal volume of isopropanol was added to the collected aqueous phase to precipitate the RNA, which was subsequently reverse transcribed to yield single-stranded cDNAs using the qPCR RT Kit (Toyobo Co., Ltd., Osaka, Japan), based on the manufacturer's instructions. The qRT-PCR reaction was performed using THUNDERBIRD qPCR Mix (Toyobo Co., Ltd.) and the ABI Prism 7300 Sequence Detection System (Applied Biosystems, Foster City, CA, USA). Each sample was analyzed in triplicate with 40 PCR cycles, each of which consisted of a denaturation step at 95°C for 15 seconds, an annealing step at 56°C for 20 seconds, and an extension step at 72°C for 40 seconds. The primer sequences used for PCR amplification in our study were designed based on the sequences of the genomic clones as follows:

SPB (175 bp: NM_147779) 5′-CTGCTTCCTACCCTCTGCTG-3′ (forward)

5′-CTTGGCACAGGTCATTAGCTC-3′ (reverse)

SPC (137 bp: NM_011359) 5′-CATCGTTGTGTATGACTACCA-3′ (forward)

5′-CCTGGAAGTTCTGGAGTTTTCT-3′ (reverse)

SPD (75 bp: NM_009160) 5′-CCTGACAAACAGAGGTGCATT-3′ (forward)

5′-GAGAAAGGGCAGCATGTCAG-3′ (reverse)

AQP5 (220 bp: NM_009701) 5′-AGAAGGAGGTGTGTTCAGTTGC-3′ (forward)

5′-GCCAGAGTAATGGCCGGAT-3′ (reverse)

GAPDH (149 bp: NM_008084) 5′-TATGTCGTGGAGTCTACTGGT-3′ (forward)

5′-GAGTTGTCATATTTCTCGTGG-3′ (reverse)

### Statistical analysis

Data were presented as the means ± standard deviation (SD). Comparison among groups was performed by the analysis of variance (ANOVA), followed by Tukey's test. *P* values less than 0.05 were considered statistically significant.

## Results

### Regulation of noncanonical Wnt signaling in mMSCs by Wnt5a, SP600125 and GF109203X

**Figure 1:**
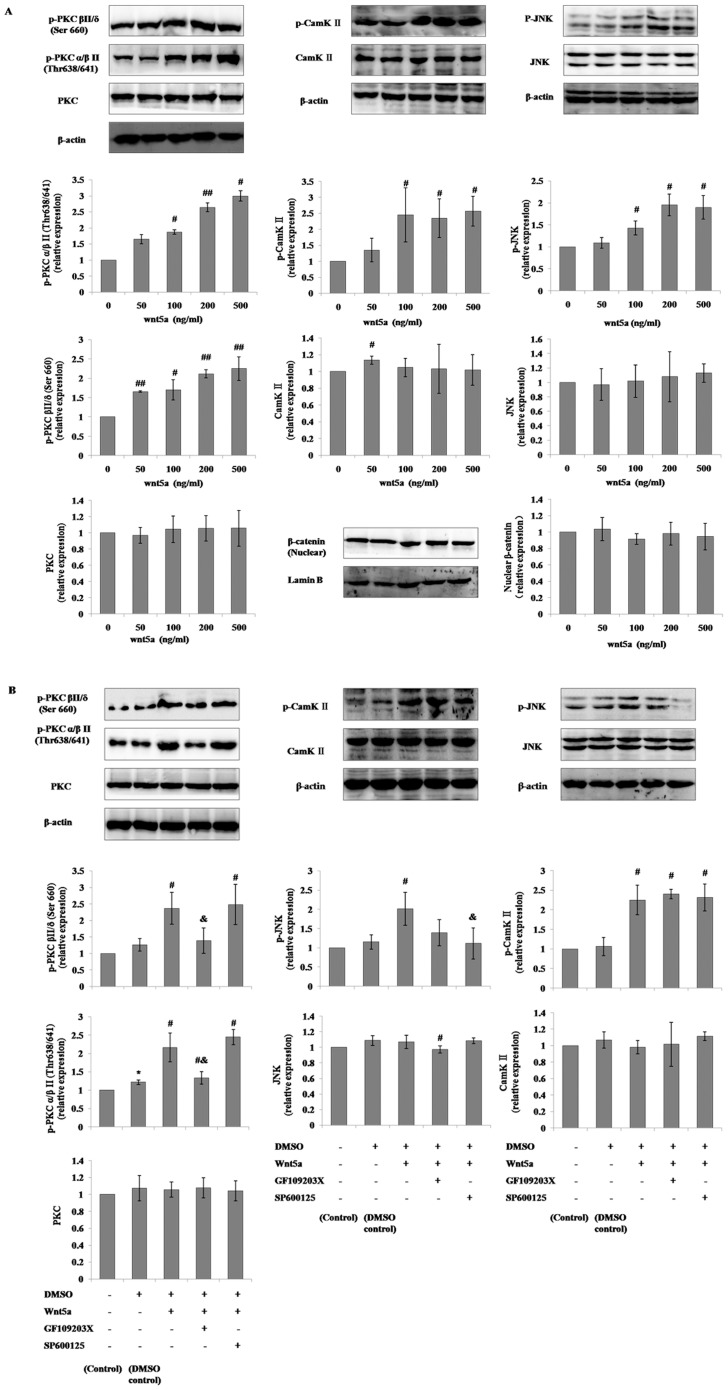
Modulation of noncanonical Wnt signaling in mMSCs with the supplementation of Wnt5a, SP600125 or GF109203X in general culture conditions. The p-PKC (pan) (β II Ser660), p-PKCα/β II (Thr638/641), PKC pan, p-SAPK/JNK (Thr183/Tyr185), SAPK/JNK, p-CamK II, CamK II β/γ/δ, and nuclear β-catenin levels in mMSCs cultured in 10% FBS-DMEM/F12 media added with different concentrations of Wnt5a (**A**) or 500 ng/ml Wnt5a plus 5 µmol/L SP600125 or 2.5 µmol/L GF109203X for 2 hours were evaluated through western blotting (**B**). (n = 3; *P<0.05 *vs* Control; #*P*<0.05, ##*P*<0.01*vs* Wnt5a 0 ng/ml or DMSO Control; &*P*<0.05 *vs* DMSO + Wnt5a + GF109203X- SP600125-).

**Figure 2:**
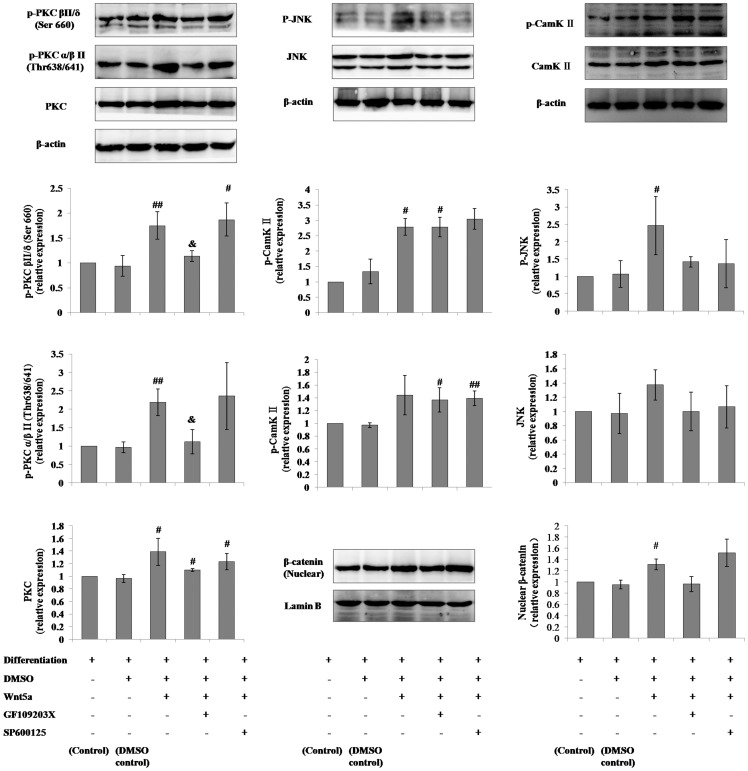
Regulation of noncanonical Wnt signaling in mMSCs with the supplementation of Wnt5a, SP600125 or GF109203X in differentiation conditions into AT II cells. The p-PKC (pan) (β II Ser660), p-PKCα/β II (Thr638/641), PKC pan, p-SAPK/JNK (Thr183/Tyr185), SAPK/JNK, p-CamK II, CamK II β/γ/δ, and nuclear β-catenin levels in mMSCs co-cultured with MLE-12 cells and SAGM with the supplementation of 500 ng/ml Wnt5a or 500 ng/ml Wnt5a plus 5 µmol/L SP600125 or 2.5 µmol/L GF109203X for 10 days were evaluated through western blotting. (n = 3; #*P*<0.05, ##*P*<0.01 *vs* DMSO control; &*P*<0.05 *vs* differentiation + DMSO + Wnt5a + GF109203X- SP600125-).

Under normal cultural conditions, phosphorylated PKC, JNK and CaMK II expression were up-regulated in a dose-dependent manner by 2-hour incubations with increasing concentrations of Wnt5a (50, 100, 200 or 500 ng/ml) and reached maximum levels after 500 ng/ml Wnt5a treatment. The PKC inhibitor, GF109203X, at 2.5 µmol/L or the JNK blocker, SP600125, at 5 µmol/L inhibited the up-regulation of phosphorylation of PKC and/or JNK caused by the 500 ng/ml Wnt5a incubation. (**Figs. 1A, 1B**) The regulatory effects of Wnt5a, SP600125 and GF109203X on the noncanonical Wnt pathway were similarly observed in mMSCs differentiated into AT II cells. (**Fig. 2**) Additionally, we investigated the effect of Wnt5a on canonical Wnt signaling through the detection of nuclear β-catenin in mMSCs by western blotting, and β-catenin was found to be elevated with the incubation of Wnt5a in mMSCs in differentiation conditions but was unchanged in mMSCs in general culture media. (**Figs. 1A, 1B** **,** **Fig. 2**)

### The noncanonical Wnt pathway was activated during the differentiation of mMSCs into AT II cells

**Figure 3:**
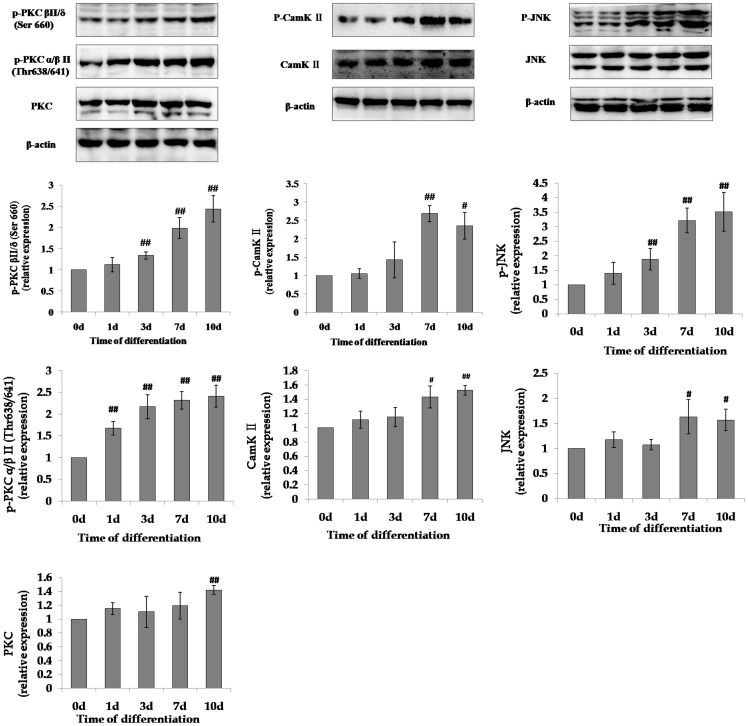
The activity of noncanonical Wnt signaling during the differentiation of mMSCs into AT II cells. The levels of the noncanonical Wnt pathway-related proteins, p-PKC (pan) (β II Ser660), p-PKCα/β II (Thr638/641), PKC pan, p-SAPK/JNK (Thr183/Tyr185), SAPK/JNK, p-CamK II, and CamK II β/γ/δ, in mMSCs on the first, third, seventh and tenth days of differentiation into AT II cells were detected through western blot. (n = 3, #*P*<0.05, ##*P*<0.01 *vs* 0 d).

According to our previous study, we drove the differentiation of mMSCs into AT II cells in an indirect co-culture system with murine lung epithelial (MLE)-12 cells plus small airway growth media (SAGM) [10]. As we confirmed before, after 10 days of differentiation, some mMSCs changed from a typical fibroblast-like spindle appearance to an epithelia-like cobblestone cell morphology. Also, lamellar body-like structures, a typical organelles of AT II cells, and numerous vacuoles were found within the cytoplasm and near the cell surface in some mMSCs after differentiation. The expression of specific markers of AT II cells, pro-SPC protein and the level of SPB, SPC and SPD mRNA in mMSCs elevated after differentiation [10]. We then examined the activation of noncanonical Wnt pathway in mMSCs during the differentiation and found that the phosphorylated and total PKC levels were significantly increased on the first, third or tenth day of differentiation of mMSCs into AT II cells, and reached their highest levels on the tenth day; the phosphorylated and total CaMK II levels were also found to be up-regulated from the seventh day, and their maximum values were observed on the seventh or tenth day. Also, the phosphorylated and total JNK levels were elevated on the third or seventh day and reached their highest levels on the tenth or seventh day, respectively. (**Fig. 3**)

### Activation of the noncanonical Wnt pathway promoted mMSCs differentiation into AT II cells

**Figure 4:**
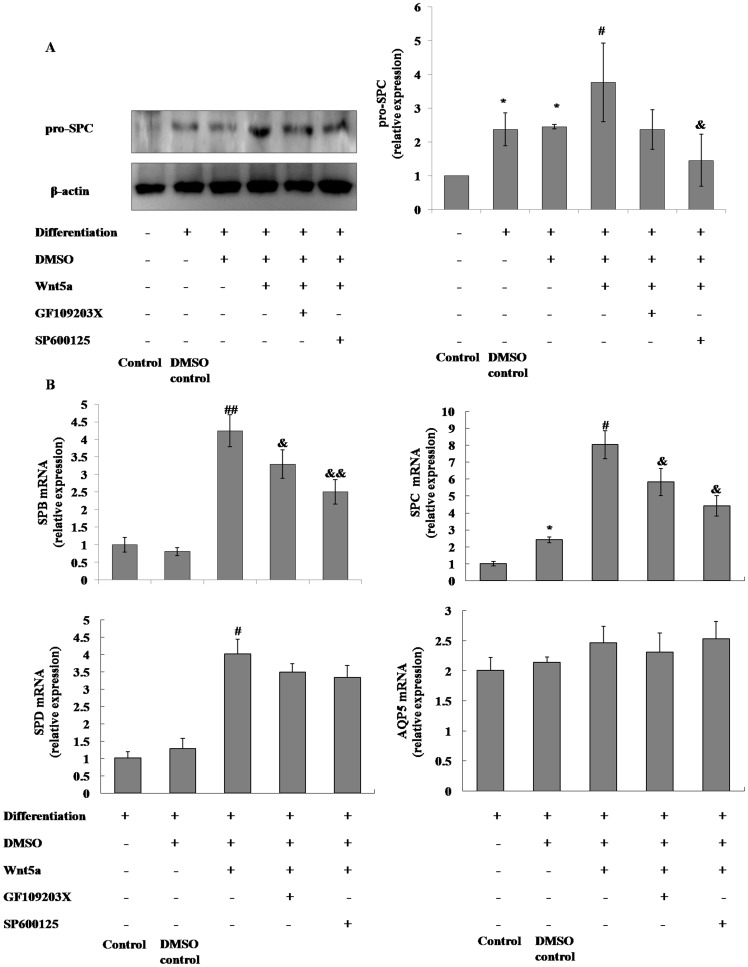
The effect of noncanonical Wnt pathway on the expression of specific markers of alveolar epithelial cells in mMSCs differentiating into AT II cells. The expression of pro-SPC protein (**A**) and SPB, SPC, SPD and AQP5 mRNA (**B**) in mMSCs after 10 days of differentiation driven by co-culture with MLE-12 cells and SAGM with supplementation of 500 ng/ml Wnt5a plus 5 µmol/L SP600125 or 2.5 µmol/L GF109203X were evaluated with western blotting and qRT-PCR. (*n* = 3; *P<0.05 *vs* Control; #*P*<0.05, ##*P*<0.01 *vs* DMSO control; &*P*<0.05, &&*P*<0.01 *vs* differentiation + DMSO + Wnt5a + GF109203X- SP600125-).

After 10 days of differentiation, the pro-SPC protein and the SPB, SPC and SPD mRNA in the mMSCs were significantly elevated after incubation with 500 ng/ml Wnt5a. Additionally, pre-incubation with either 5 µmol/L SP600125 or 2.5 µmol/L GF109203X before the 500 ng/ml Wnt5a treatment reversed the promotion of Wnt5a on pro-SPC protein and SPB and SPC mRNA, and SP600125 seemed to have a greater inhibitory effect than GF109203X treatment. However, Wnt5a, SP600125 and GF109203X had no effect on the expression of AQP5 mRNA. (**Fig. 4**)

### Activation of the noncanonical Wnt pathway had no significant effect on the proliferation of mMSCs

**Figure 5:**
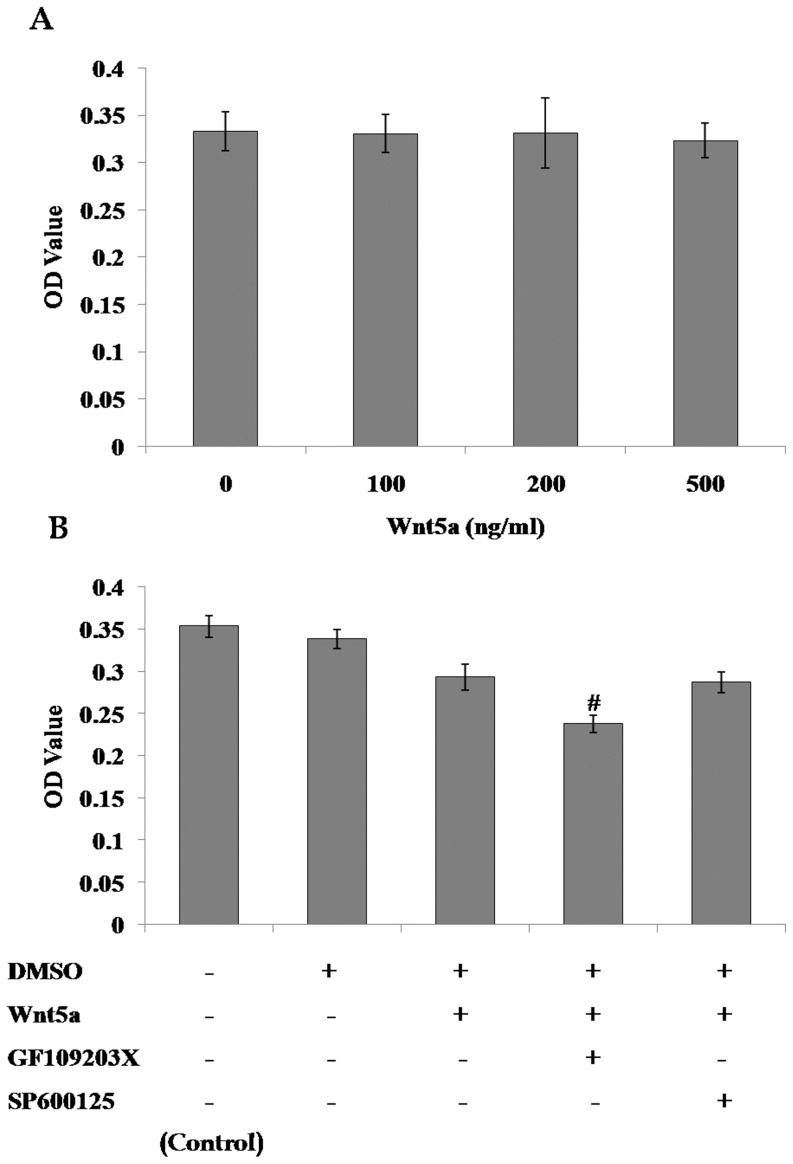
Role of noncanonical Wnt signaling in the proliferation of mMSCs. The proliferation of mMSCs was evaluated using MTT assay after incubation in 2% FBS-DMEM/F12 media supplemented with increasing concentrations of Wnt5a (**A**) or 500 ng/ml Wnt5a plus 5 µmol/L SP600125 or 2.5 µmol/L GF109203X (**B**) for 72 h. (*n* = 4; #*P*<0.05 *vs* Control).

The effect of activation of the noncanonical Wnt pathway on mMSC proliferation was evaluated using MTT assays after incubation for 3 days with increasing concentrations of Wnt5a (100, 200, or 500 ng/ml), and no significant differences were observed after 100–500 ng/ml Wnt5a intervention. Interestingly, the supplementation of GF109203X plus Wnt5a reduced the number of mMSCs. (**Figs. 5A, 5B**)

### Effect of cellular toxicity from oxidative stress on viability and noncanonical Wnt signaling in mMSCs

**Figure 6:**
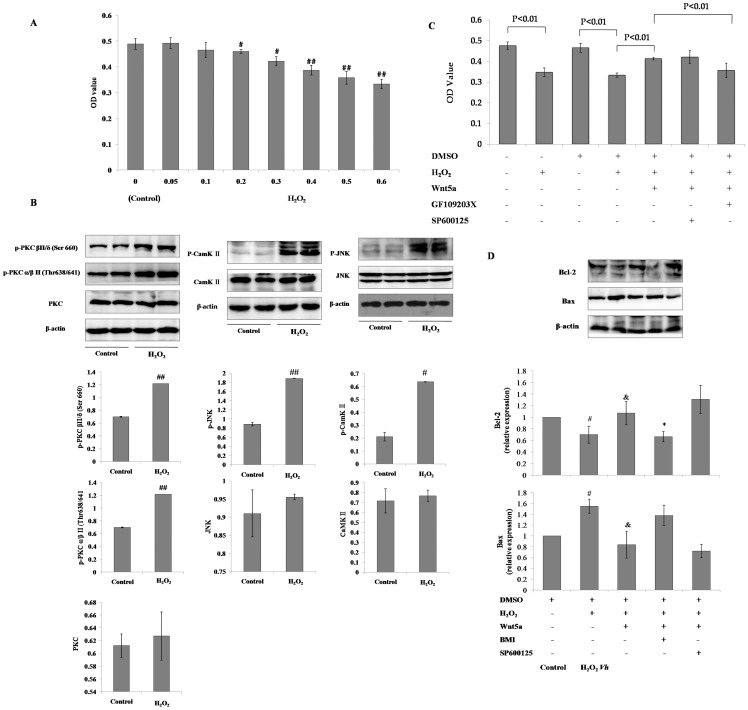
Role of noncanonical Wnt pathway in the H_2_O_2_-induced cellular toxicity of mMSCs. The viability of mMSCs after treatment with increasing concentrations of H_2_O_2_ supplemented in 2% FBS-DMEM/F12 growth media for 12 hours was determined using an MTT assay (**A**, *n* = 4). The expressions of p-PKCα/β II (Thr638/641), p-PKC (pan) (β II Ser660), p-SAPK/JNK (Thr183/Tyr185), p- CamK II, PKC pan, SAPK/JNK, CamK II β/γ/δ in mMSCs with 0.2 mmol/L H_2_O_2_ treatment for 12 hours were analyzed using western blotting (**B**, *n* = 3). The effect of pretreatment mMSCs with 500 ng/ml Wnt5a plus 5 µmol/L SP600125 or 2.5 µmol/L GF109203X for 1 hour on mMSC survival (**C**, *n* = 4) and the expression of Bcl-2 and Bax (**D**, *n* = 3) influenced by 0.2 mmol/L H_2_O_2_ for 12 hours were analyzed using MTT assay and western blotting. (#*P*<0.05, ##*P*<0.01 *vs* control, &*P*<0.05 *vs* H_2_O_2_ *Vh*, *P<0.05 *vs* DMSO+ H_2_O_2_+ Wnt5a- SP600125- GF109203X-).

In the present study, oxidative stress in mMSCs was induced with H_2_O_2_. Increasing H_2_O_2_ concentrations (0.05, 0.1, 0.2, 0.3, 0.4, 0.5 and 0.6 mmol/L) reduced the viability of mMSCs in a concentration-dependent manner after 12 hours of incubation, as evaluated using the MTT assay (**Fig. 6A**). The minimum concentration of H_2_O_2_ that significantly inhibited viability, 0.2 mmol/L, was chosen for subsequent experiments. We then evaluated the expression of the apoptosis-related proteins Bax and Bcl-2 in mMSCs incubated with 0.2 mmol/L H_2_O_2_. We found that the Bcl-2/Bax ratio in mMSCs exposed to 0.2 mmol/L H_2_O_2_ assessed by western blotting analysis was significantly reduced compared to control (**Fig. 6D**). Additionally, noncanonical Wnt pathway-related proteins, including p-JNK, p-PKC and p-CaMK II, were all up-regulated in mMSCs after H_2_O_2_ treatment (**Fig. 6B**).

### Activation of noncanonical Wnt pathway-protected mMSCs from oxidative stress-induced injury

mMSCs pre-incubated with 500 ng/ml Wnt5a for one hour offered significant protection against cell death induced by 12 hours of 0.2 mmol/L H_2_O_2_ incubation (**Fig. 6C**). Correspondingly, the decreased Bcl-2/Bax ratio in the H_2_O_2_-treated mMSCs was reversed by the activation of the noncanonical Wnt pathway by Wnt5a. Supplementation with GF109203X significantly reversed the effect of Wnt5a, but supplementation with SP600125 showed no significant difference from the H_2_O_2_ vehicle (**Fig. 6D**).

### Activation of the noncanonical Wnt pathway-promoted migration of mMSCs

**Figure 7:**
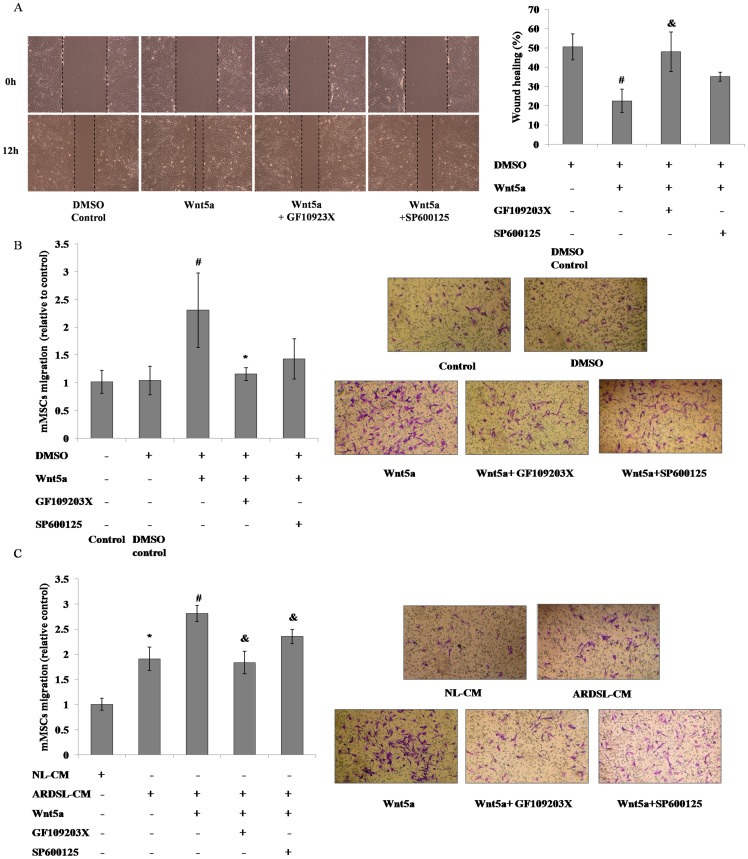
Role of noncanonical Wnt pathway in the migration of mMSCs. The horizontal migration of mMSCs incubated in 2% DMEM/F12 media supplemented with 500 ng/ml Wnt5a or 500 ng/ml Wnt5a plus 2.5 µmol/L GF109203X or 5 µmol/L SP600125 was examined by wound healing assay (**A** ×200; *n* = 3; #*P*<0.05 *vs* DMSO control; &*P*<0.05 *vs* DMSO+ Wnt5a+ GF109203X- SP600125-). The vertical migration of mMSCs incubated in 2% DMEM/F12 media supplemented with 500 ng/ml Wnt5a or 500 ng/ml Wnt5a plus 2.5 µmol/L GF109203X or 5 µmol/L SP600125 towards 10% FBS-DMEM/F12 media (10% FBS-GM) (**B** ×200; *n* = 3; #*P*<0.05 *vs* DMSO control; *P<0.05 *vs* DMSO+ Wnt5a+ GF109203X- SP600125-) or conditioned media from normal (NL-CM) or ARDS mouse-derived lung tissue (ARDSL-CM) (**C** ×200; *n* = 3; *P<0.05 *vs* NL-CM; #*P*<0.05 *vs* ARDSL-CM; &*P*<0.05 *vs* ARDSL-CM+ Wnt5a+) was examined through Transwell inserts migration assay.

Compared to control, supplementation with 500 ng/ml Wnt5a significantly reduced the gap of the wound in the wound healing assay and enhanced the vertical migration of mMSCs towards the higher concentration of FBS in the lower chambers in the Transwell inserts assay. However, the Wnt5a effect was significantly blocked by GF109203X and partly inhibited by SP600125 (**Figs. 7A, 7B**). We subsequently examined mMSCs migration to conditioned media from normal or ARDS mice-derived lung tissue in the lower chambers of the Transwell inserts and the influence of noncanonical Wnt pathway activation on this process. We found that ARDS lung tissue attracted more mMSCs than normal lung tissue and the migration of mMSCs towards the conditioned medium of ARDS mice-derived lung tissue was further enhanced with Wnt5a, whose effect was reversed with the incubation of SP600125 or GF109203X (**Fig. 7C**).

### Higher levels of noncanonical Wnt ligands in lung tissue of ARDS mice

**Figure 8:**
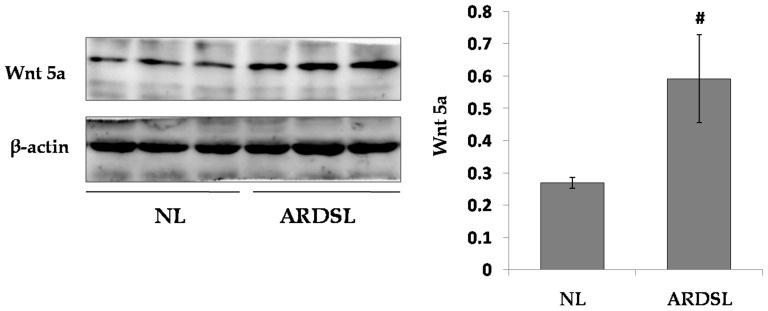
Wnt5a ligand in lung tissue of normal or ARDS mice. The expression of Wnt5a in normal or ARDS mouse-derived lung tissue was evaluated through western blotting analysis. (*n* = 3; #*P*<0.05 *vs* NL).

To explore the levels of noncanonical Wnt ligands in normal or inflammatory lung tissue, Wnt5a in lung tissue was analyzed via western blotting. We found a significantly higher expression of Wnt5a in ARDS mouse-derived lung tissues than in normal ones. (**Fig. 8**)

## Discussion

AT II cells are considered critical for the repair of injured lung tissue in ARDS patients [2], [17]. Many investigations, including our previous study, have disclosed that multipotent MSCs could differentiate into AT II cells in ARDS animals and in some *in vitro* conditions [6]–[10], [18]. These findings partly contribute to the efficacy of MSCs in ARDS treatment. Elucidation of the mechanisms underlying the differentiation of mMSCs into AT II cells, which has not yet been fully explored, may promote the development of ARDS therapy. The role of the canonical Wnt pathway in the differentiation of mMSCs into AT II cells has been elucidated in our previous study [10]; however, the effect of the noncanonical Wnt pathway, which transduces a signal independent of the accumulation of β-catenin, in this process has little been discussed. In the present study, we found that the noncanonical Wnt signaling pathway was involved in the differentiation of mMSCs into AT II cells in vitro and that the activation of the Wnt/PCP (Wnt/JNK) or Wnt/Ca^2+^/PKC pathway with Wnt5a favored the differentiation of MSCs into AT II cells in a co-culture system.

As we know, about nine pathways in the transduction of noncanonical Wnt signaling have been disclosed, although they have not yet been well described. Wnt/PCP (or Wnt/JNK) and Wnt/Ca^2+^ signaling are the two most studied molecular signaling pathways. The Wnt/JNK pathway is initiated by the activation of FZD and Ror2 with Wnts [11], [12]. This signal is transduced through Dvl and Ras homologue GTPases (RhoGTPases) to activate the nuclear transcription factor activator protein 1 (AP-1), which is composed of proteins of the Jun family (e.g., c-Jun, Jun-B, Jun-D), Fos family (e.g., c-Fos, foxB, Fra-1, Fra-2), ATF family (e.g., ATF-α, ATF-2, ATF-3) and JDP family (e.g., JDP-1, JDP-2). The activation of AP-1 is critical for many biological behaviors, including cell differentiation, proliferation, and apoptosis [19]–[21]. In the Wnt/Ca^2+^ pathway, the binding of the Wnts to their receptors, including Frz2, 3, 4 and 5, increases the intracellular release of Ca^2+^ or the extracellular Ca^2+^ influx, which then activates PKC and CaMK II. It was found that PKC could regulate cell adhesion and tissue development during the development of vertebrates [22], [23].

Through the activation of PKC, CaMK II and JNK, the noncanonical Wnt pathway plays important roles in diverse biological behaviors of cells and organisms that are different from the roles of the canonical Wnt pathway. Many ligands and receptors belonging to this pathway are expressed in MSCs and can influence the biological functions of MSCs, including migration, differentiation, and development. He et al. [24] found that Wnt11 could promote MSC differentiation into myocardial cells via the noncanonical Wnt pathway. A similar effect of Wnt4 on the differentiation of MSCs into osteoblasts in vitro and in vivo was observed in the study by Chang et al. [25]. Diverse effects of noncanonical Wnt signaling on the differentiation of MSCs, according to the target cell of differentiation, were observed in other studies. Some studies showed that the up-regulation of Wnt/JNK signaling inhibited adipogenesis while stimulating osteoblastogenesis in MSCs. Furthermore, even for the same type of target cells, the developmental stage of the target cells can also influence the effect of the noncanonical Wnt pathway. A study by Bergwitz, et al. [26] suggested that Wnt4 could inhibit chondrocyte differentiation while favoring late maturation. Topol, et al. [15] also found that the opposing noncanonical signals of Wnt5a hinder the chondrogenic lineage commitment of MSCs, promote chondrocyte differentiation, and delay chondrocyte maturation into hypertrophic stages, but their effects on the differentiation of MSCs into AT II cells have not been explored.

In our study, we firstly examined the regulation of noncanonical Wnt signaling in mMSCs with Wnt5a, a specific ligand that activates the noncanonical Wnt pathway and GF109203X, an inhibitor of PKC, or SP600125, an inhibitor of JNK. Interestingly, GF109203X was found to inactivate JNK in MSCs while SP600125 did not influence the activation of PKC, suggesting that phophorylated PKC might be the upstream effector of JNK; this finding was in accordance with other studies [27], [28]. Additionally, crosstalk between canonical and noncanonical Wnt signaling has been shown previously. It was found that Wnt/Ca^2+^ signaling could activate the TAK1-NLK MAPK pathway, which might reduce the transcription of TCF/LEF 1 without influencing the accumulation of β-catenin, thus, inhibiting canonical Wnt signaling [29], [30]. However, some recent studies suggested that noncanonical Wnt signaling also could stimulate the canonical Wnt pathway and the effect of noncanonical Wnt ligands on the activation of canonical Wnt signaling might be related to the kind of FZD receptors on the target cells [31], [32]. In the present study, no significant difference in the nuclear accumulation of β-catenin was observed after 2 hours of incubation with Wnt5a, whereas a rised accumulation of β-catenin was found after 10 days of treatment with Wnt5a in the differentiation conditions to some extent. There might be some unclear signaling mechanisms in the differentiation, which may need more investigations.

Like the canonical Wnt pathway, the noncanonical Wnt pathway-related proteins are expressed in embryonic and adult lung tissues and are involved in physiological and pathological processes of the lung [33]. The overexpression of Wnt5a in transgenic mice and in chicks has been reported to result in severe pulmonary hypoplasia. [34]. Increasing the downregulated noncanonical Wnt receptor Fzd2 with GATA 6 mutants was found to be able to partly reduce the lung epithelial defects induced by GATA6 mutants [35]. In addition to their physiological effects, the noncanonical Wnt pathway also participates in the carcinogenesis and invasion of lung cancer [36], [37]. In the present study, the upregulation of Wnt5a was also observed in lung tissue of ARDS mice induced with LPS through intratracheal administration. Therefore, the noncanonical Wnt pathway is hypothesized to be involved and potentially effective in the differentiation of MSCs into pulmonary cells.

To examine the role of the noncanonical Wnt pathway in the differentiation of MSCs into AT II cells, a co-culture condition with MLE-12 cells plus SAGM, verified to be effective at driving the differentiation of MSCs into AT II cells in our previous study, was used in the present study [10]. As the phosphorylation levels of JNK, PKC, and CaMK II were increased in the third to tenth days of co-culture, the noncanonical Wnt pathway was considered to be involved in the differentiation process. Further supplementation of Wnt5a increased the expression of the pro-SPC protein and the SPB, SPC and SPD mRNA, and this effect could be inhibited with the addition of either the PKC or JNK inhibitors. As phophorylated PKC might be an upstream effector of JNK, we were not able to elucidate whether Wnt/JNK alone or both Wnt/JNK and Wnt/PKC are the signaling pathways that regulate the differentiation. Either PKC or JNK was reported to be involved in regulating the differentiation of the noncanonical Wnt pathway. Yu, et al. found that the inactivation of PKC could reverse the promotion of differentiation into neural progenitor cells by Wnt5a [38]. A similar suppression effect of Wnt/PKC signaling was also observed in the investigation of the pro-differentiation of circulating progenitor cells into cardiomyogenic cells [39]. In another study, Wnt2 activating the noncanonical Wnt pathway through JNK/AP1 signaling induced the differentiation of embryonic stem cells into cardiac myocytes [40]. In the investigation by Qiu, et al, the activation of Wnt/JNK signaling by anisomycin enhanced osteoblast differentiation, whereas its inhibition by SP600125 enhanced adipocyte differentiation of human MSCs [41]. Some studies have reviewed the importance of both JNK and PKC in the pro-differentiation effect of the noncanonical Wnt pathway [42].

The migration and concentration of MSCs into injured or inflammatory sites after administration *in vivo* occurs prior to the exertion of their biological functions, including an anti-inflammation function, specialized cell-type differentiation and repair of injured tissues. Several studies have observed an enhanced recruitment of transplanted MSCs to the injured lung tissue in ARDS mice compared to the recruitment in control mice [43]. Some potential molecular mechanisms, including the cell derived factor-1α (SDF-1α) and its cellular receptor CXCR4, canonical Wnt signaling and so on, that drive the migration of MSCs have been presented in several previous studies [10], [44]–[47]. A role for noncanonical Wnt signaling in the migration of endothelial cells, gastric cancer cells, pancreatic cancer cells, and breast cancer cells, among others, has been shown by recent studies [48]–[51]; however, its effect on the migration of mMSCs has not been thoroughly explored. Our results showed that the activation of the noncanonical Wnt pathway through the incubation of mMSCs with Wnt5a significantly increased the migration of the mMSCs and blockers of either PKC or JNK could decrease or block the enhancement of migration caused by Wnt5a. The promotion of Wnt/PCP signaling in the migration of MSCs might be mediated through the up-regulation of some receptor of chemokines. Jin, et al. found that the inactivation of JNK led to the reduced expression of CXCR4 in Ewing sarcoma cells upregulated by Wnt5a [52]. In other investigations, JNK, through its downstream transcriptional factor AP-1, upregulated metalloproteinase (MMP)-9 or the phosphorylation of paxillin to regulate the migration of cells [53], [54]. The activation of MMP-9 and the regulation of the cytoskeleton might also be the underlying mechanisms for the enhanced migration induced by Wnt/PKC signaling [55]. In our present study, it was hard to tell whether the Wnt/PKC and Wnt/JNK pathways or the Wnt/JNK pathway alone mediated the enhancement of migration by Wnt5a in mMSCs.

The proliferation and survival of MSCs in the injured tissues after transplantation also played an important role in their differentiation to the target cells. Cell numbers in the target tissue decreased rapidly after the administration of MSCs [56]. Mei et al. [57] found that approximately 47% of MSCs injected into the lung tissue of ARDS mice 15 minutes after induction of ARDS decreased to less than 8% 3 days later. The low survival of grafted MSCs limits their transdifferentiation and effects on target tissue repair [58]. Both the excitatory and inhibitory effects of the noncanonical Wnt pathway on cell proliferation have been previously reported. Some studies found that Wnt5a could increase the proliferation of fibroblasts or endothelial cells [59], while some investigators insisted that Wnt5a had a negative effect on human endothelial cell proliferation [60]. In our present study, no significant difference in the proliferation of mMSCs was observed after Wnt5a treatment compared to the control. The effect of Wnt5a on the mMSC proliferation might need further exploration.

There are several factors in complex *in vivo* situations that negatively influence the survival of transplanted MSCs. Oxidants, which are produced from inflammatory lung tissue and are a causative factor of lung injury [61], are detrimental to MSCs transplanted *in vivo* and trigger their apoptosis [62]. Hydrogen peroxide (H_2_O_2_), a typical oxidant, was adopted in our study to induce oxidative damage to mMSCs. We found a H_2_O_2_ dose-dependent decrease in the survival of mMSCs and the down-regulation of Bcl-2/Bax, the balance of which determines whether a cell undergoes apoptosis [63]. The activation of the noncanonical Wnt pathway in mMSCs by H_2_O_2_ *in vitro* was observed in our study. Vuga, et al. found that Wnt5a intervention could enhance resistance to apoptosis caused by H_2_O_2_ [64], but the role of the noncanonical Wnt pathway in the injury of mMSCs induced by H_2_O_2_ has not yet been explored. In our study, the supplement of Wnt5a prior to H_2_O_2_ treatment reversed the decline of survival and the Bcl-2/Bax ratio in mMSCs induced by H_2_O_2_ treatment. Interestingly, no significant difference was found after SP600125 intervention, and a reverse in the deterioration induced by H_2_O_2_ was detected with GF109203X treatment. It appeared that Wnt5a, through Wnt/PKC signaling, mediated the protection against the damage of mMSCs induced by H_2_O_2_. This finding was in accordance with the results of the investigation of Bluwstein et al [65]. JNK was found in some other studies to participate in cell death, and the inhibition of JNK activation ameliorated the H_2_O_2_ induced apoptosis [66]–[68].

## Conclusions

Our results demonstrated the importance of the noncanonical Wnt pathway in the differentiation of mMSCs into AT II cells in a co-cultured system with MLE-12 cells and SAGM. Wnt5a, either through Wnt/JNK signaling alone or through the combination of Wnt/JNK and Wnt/PKC signaling, promoted the differentiation of mMSCs into AT II cells and their migration towards ARDS lung tissue. Furthermore, Wnt5a supplementation, through Wnt/PKC signaling, increased the survival of mMSCs after being treated with H_2_O_2_ *in vitro* but had no significant influence on the proliferation of mMSCs. From these results, we speculate that noncanonical Wnt signaling plays a critical role in the differentiation of mMSCs into pneumonocytes and in the repair of injured lung tissue *in vivo*, and this needs to be confirmed by future studies.
